# ENHANCE proof-of-concept three-arm randomized trial: effects of reaching training of the hemiparetic upper limb restricted to the spasticity-free elbow range

**DOI:** 10.1038/s41598-023-49974-6

**Published:** 2023-12-22

**Authors:** Mindy F. Levin, Sigal Berman, Neta Weiss, Yisrael Parmet, Melanie C. Baniña, Silvi Frenkel-Toledo, Nachum Soroker, John M. Solomon, Dario G. Liebermann

**Affiliations:** 1 Faculty of Medicine and Health Sciences, School of Physical and Occupational Therapy, McGill University, 3654 Promenade Sir William Osler, Montreal, QC H3G 1Y5 Canada; 2 Center for Interdisciplinary Research in Rehabilitation (CRIR), Montreal, QC Canada; 3 Department of Industrial Engineering and Management, Ben-Gurion University of the Negev, Beer-Sheva, Israel; 4 The Zlotowski Center, Ben-Gurion University of the Negev, Beer-Sheva, Israel; 5 Department of Physical Therapy, Ariel University, Ariel, Israel; 6 Department of Neurological Rehabilitation, Loewenstein Rehabilitation Hospital, Ra’anana, Israel; 7 Faculty of Medicine, Tel Aviv University, Tel Aviv, Israel; 8 Department of Physiotherapy, Manipal College of Health Professions, Manipal Academy of Higher Education, Manipal, Karnataka India; 9 Centre for Comprehensive Stroke Rehabilitation and Research, Manipal Academy of Higher Education, Manipal, Karnataka India; 10 Department of Physical Therapy, Faculty of Medicine, Stanley Steyer School of Health Professions, Tel Aviv University, POB 39040, 61390 Ramat Aviv, Tel Aviv, Israel

**Keywords:** Translational research, Neurological disorders, Spinal cord, Stroke

## Abstract

Post-stroke motor recovery processes remain unknown. Timescales and patterns of upper-limb (UL) recovery suggest a major impact of biological factors, with modest contributions from rehabilitation. We assessed a novel impairment-based training motivated by motor control theory where reaching occurs within the spasticity-free elbow range. Patients with subacute stroke (≤ 6 month; n = 46) and elbow flexor spasticity were randomly allocated to a 10-day UL training protocol, either personalized by restricting reaching to the spasticity-free elbow range defined by the tonic stretch reflex threshold (TSRT) or non-personalized (non-restricted) and with/without anodal transcranial direct current stimulation. Outcomes assessed before, after, and 1 month post-intervention were elbow flexor TSRT angle and reach-to-grasp arm kinematics (primary) and stretch reflex velocity sensitivity, clinical impairment, and activity (secondary). Results were analyzed for 3 groups as well as those of the effects of impairment-based training. Clinical measures improved in both groups. Spasticity-free range training resulted in faster and smoother reaches, smaller (i.e., better) arm-plane path length, and closer-to-normal shoulder/elbow movement patterns. Non-personalized training improved clinical scores without improving arm kinematics, suggesting that clinical measures do not account for movement quality. Impairment-based training within a spasticity-free elbow range is promising since it may improve clinical scores together with arm movement quality.

Clinical Trial Registration: URL: http://www.clinicaltrials.gov. Unique Identifier: NCT02725853; Initial registration date: 01/04/2016.

## Introduction

Stroke is a leading cause of long-term sensorimotor disability, including persistent deficits in upper limb (UL) function^1^. Understanding how to improve UL recovery is a major challenge^2^ yet, despite numerous studies based on established neurorehabilitation principles, post-stroke UL recovery remains incomplete^1,3^ with up to 62% of stroke survivors showing sensorimotor deficits for more than 6 months^4^.

**Figure 1:**
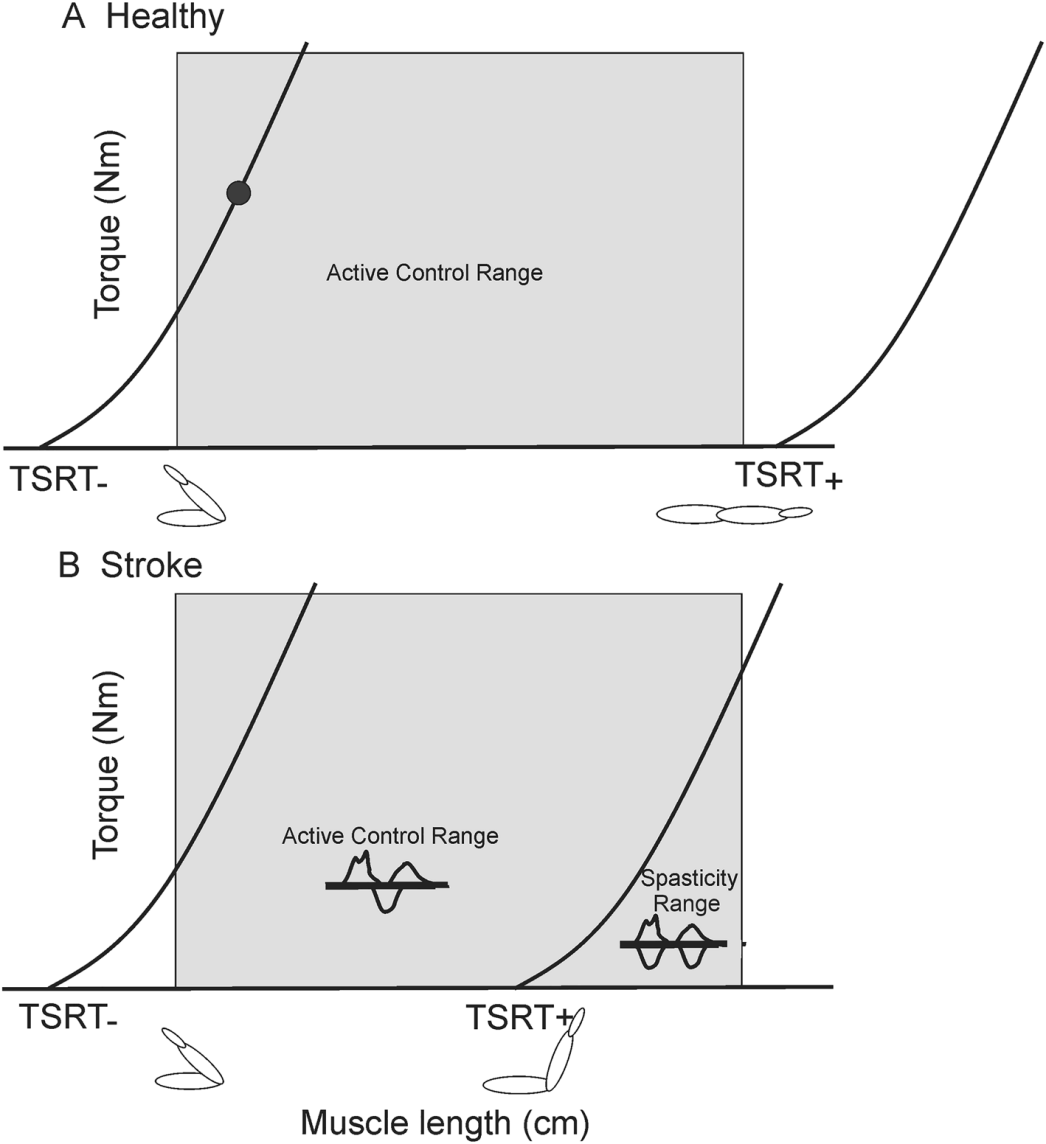
Tonic stretch-reflex thresholds (TSRTs) in healthy and stroke participants. (**A**) The biomechanical joint range of the elbow joint (grey shaded area) extends from full joint flexion to full joint extension. To produce muscle activation, the central nervous system specifies the threshold for muscle activation (TSRT) and its associated torque-length relationship (curved line). To obtain high torques at short muscle lengths, the TSRT has to be regulated beyond the lower biomechanical limit (TSRT_ and left curved line). The distance between the actual muscle length (solid circle) and the referent length (i.e., the TSRT_) defines the EMG level. To achieve full muscle relaxation throughout the joint range, the TSRT and torque-length relationship must be regulated beyond the upper biomechanical limit (TSRT_+_ and right curved line). The range of regulation of TSRT (TSRT_ to TSRT_+_) extends beyond the biomechanical range to permit the establishment of any level of muscle activation at any muscle length (Active Control Range). (**B**) Stroke results in the limitation of the regulation of TSRT_+_ such that, at rest, the TSRT_+_ defines the muscle length at which spasticity begins to appear (Spasticity Range). In the Active Control Range between TSRT_ and TSRT_+_, normal reciprocal muscle activation (inset) during slow active extension is possible. Slow voluntary extension movements beyond TSRT_+_ are characterized by abnormal muscle coactivation (inset).

Current rehabilitation therapies do not enhance UL recovery beyond the effects of biological factors that drive brain plasticity towards adaptive re-mapping and re-wiring, mainly in the early sub-acute period^5^. However, recent research suggests that patients with chronic stroke could adapt elbow movements to a sudden addition/withdrawal of an elastic load significantly better when elbow extension was restricted to a spasticity-free range where active control of muscle activation was possible^6^. This suggests that in that range, the lesioned sensorimotor system can still exert some control over muscle activation thresholds (i.e., the Tonic Stretch Reflex Threshold—TSRT). In the healthy nervous system, to ensure muscle relaxation at rest, the central nervous system (CNS) sets the TSRT outside of the biomechanical range (Fig. 1A, TSRT_+_). To activate the muscle, the CNS sets the TSRT at a desired joint angle within or beyond the range (‘active control range’, Fig. 1A, TSRT_−_^7^). After stroke, however, TSRT control is impaired such that TSRT_+_ lies abnormally within the biomechanical range at rest resulting in reflex-mediated muscle activation at muscle lengths beyond this threshold and disruption of voluntary muscle activation patterns (i.e., “spasticity range”; Fig. 1B,^8,9^). At shorter muscle lengths, normal reciprocal muscle activation is relatively preserved, thus accounting for near-normal error-correction strategies within this range (i.e., “active control range”)^6,9^. Support for the role of TSRT regulation within the angular joint range on motor impairment derives from several studies^10–12^ and the observation of preserved modulation of motor evoked-potential amplitude in elbow flexors of post-stroke patients when the limb was positioned within the TSRT-defined active control range but not within the spasticity range^13^.

Personalized impairment-based UL training, tuned to the spatial structure of the individual’s UL motor deficits, was combined with technologies shown to improve UL sensorimotor recovery by maximizing motor learning. Practice of reaching tasks was done in a Virtual Reality (VR) environment, while receiving excitatory anodal-tDCS (a-tDCS) over sensorimotor areas of the affected hemisphere. VR offers motivating practice environments incorporating activities important for neurological rehabilitation, such as goal‐oriented tasks, repetition, and feedback^14^. In addition, a-tDCS was used to enhance effects based on restoring inter-hemispheric balance disrupted after stroke^15^.

We originally hypothesized that, in patients with sub-acute post-stroke spastic hemiparesis, repetitive reaching practice restricted to elbow extension ranges that did not evoke elbow flexor spasticity (personalized training) combined with excitatory a-tDCS, would improve the range of spasticity-free active elbow extension (i.e., increase the resting TSRT angle) and UL sensorimotor outcomes more than training with a-tDCS or personalized training alone. We also hypothesized that an increase in the resting TSRT angle would be related to improved kinematics of a standardized reaching task and clinical outcomes.

However, since there were no differences in the main effects of treatment range with or without a-tDCS (see Results, Table 1), we conducted a secondary analysis on participants who practiced reaching in the Restricted-range versus the Non-restricted-range. This analysis focused on whether training the hemiparetic UL restricted to the individually determined spasticity-free range during reaching movements would lead to greater gains than whole-range reaching training. We hypothesized that personalized training in the spasticity-free range would lead to (1) an increased elbow-flexor TSRT angle (i.e., greater active control range) associated with (2) improved kinematics, (3) decreased sensitivity of the stretch reflex (μ), and (4) improved clinical outcomes compared to arm reaching training without elbow-range restrictions.

**Table 1:** Descriptive data: Mean (SD) values for pre-test scores for demographic and initial clinical scores of all treatment conditions according to the three original groups and to the groups analyzed according to training range.

Variables	Groups				
	Group 1 (n = 16)	Group 2 (n = 15)	Group 3 (n = 15)	Restricted Range (n = 25)	Non-Restricted Range (n = 10)
Age (years)	53.6 (11.4)	51.1 (17.8)	52.3 (16.9)	54.2 (11.9)	52.3 (9.4)
Male/female (#)	10/6	9/6	10/5	17/8	8/2
Side of hemiparesis (L/R, #)	7/9	7/8	7/8	13/12	4/6
Time since stroke event (days)	64.6 (44.0)	65.9 (29.6)	81.4 (54.5)	70.1 (49.6)	60.5 (21.7)
Lesion location (# ischemic/hemorrhagic) (%)	94 / 6	73 / 27	67 / 33	76 / 24	60 / 40
MOCA (/30 pt)	29.4 (4.5)	23.3 (5.8)	24.9 (4.8)	25.5 (4.4)	26.0 (4.8)
MAS – elbow flexors (/4pt)	1.44 (0.51)	1.43 (0.26)	1.43 (0.26)	1.42 (0.40)	1.45 (0.16)
FMA (/66 pt)	37.3 (13.0)	28.9 (13.7)	32.5 (9.6)	34.9 (12.3)	26.7 (13.7)
WMFT – FAS (pt)	2.80 (1.01)	2.30 (1.22)	2.85 (0.74)	2.84 (0.94)	2.22 (1.19)
TSRT (deg)	113.2 (21.2)	88.4 (21.8)*	100.7 (4.5)	105.1 (21.0)	92.9 (24.0)
μ (s)	0.073 (0.064)	0.041 (0.039)	0.059 (0.038)	0.088 (0.082)	0.066 (0.091)
Training (total time, min)	459.9 (92.4)	388.8 (142.8)	458.8 (110.1)	463.9 (82.9)	432.8 (143.6)
Training intensity (mean #reps/min)	8.1 (2.9)	6.2 (1.9)	7.8 (3.0)	7.4 (2.3)	5.5 (1.4)
Other therapies (mean min/day)	79.9 (10.2)	71.3 (7.3)	75.4 (5.1)	79.9 (10.2)	71.3 (7.3)

FMA, Fugl-Meyer Assessment; MAS, Modified Ashworth Scale; L, Left; MOCA, Montreal Cognitive Assessment; NA, Not Applicable; R, Right; TSRT, Tonic Stretch Reflex Threshold; WMFT-FAS, Wolf Motor Function Test-Functional Activity Score.

*Difference between Group 1 and Group 2 ANOVA post-hoc deviation F2,1 = 8.496, *p* = 0.006.

## Methods

### Study design and participants

A single-blind proof-of-concept three-arm randomized trial was conducted on hospitalized patients in three countries (Canada/Israel/India) between July 2016 and March 2020. Research ethics approval was obtained from each center. The study protocol is available^16^. Inclusion criteria for patients with mild-to-moderate symptoms were: 1) first-ever stroke in middle cerebral artery territory, confirmed by MRI/CT, 3 weeks to 6 months previously to enhance the homogeneity of the study population; 2) aged 25–80 yr; 3) medically stable; 4) arm paresis: Chedoke-McMaster Stroke Assessment (CMSA^17^) 2–6/7; 5) minimum 30° active elbow flexion and extension; 6) elbow flexor spasticity: determined by TSRT measurement or Modified Ashworth Scale (MAS^18^ score > 1 + /4 score. Exclusion criteria were: 1) unstable medical condition; 2) major cognitive deficits (˂ 20 on Montreal Cognitive Assessment (MoCA)^19^; 3) history of neurological/psychiatric disorders, alcohol/drug abuse, seizures, migraines, metal in cranium, cochlear or cardiac implants; 4) taking anti-epileptic or psychoactive medications^20^. Participants provided written informed consent based on the Declaration of Helsinki. Participants were randomly allocated by a blinded research assistant (permuted block randomization stratified for age [25–50 yr/51–80 yr] and chronicity [3–12 wk/13–42 wk]) to one of three groups using opaque envelopes. Intervention therapists were blinded to assessment outcomes. Evaluators were blinded to group assignments (i.e., single-blind: patients not blinded as to their group assignment).

### Procedures

Identical training (VR: Jintronix, Inc. Montreal, Canada) and data collection (TSRT: Montreal Spasticity Measure; Kinematics: Polhemus electromagnetic system, Colchester, VT, USA) equipment was provided to each research site to minimize discrepancies due to training or evaluation procedures across sites. Personnel at each site also received standardized training on study procedures. Participants were randomly assigned to one of 3 groups and engaged in 10 treatment sessions delivered over 2 weeks (5 days/wk). Group 1 practiced personalized reaching training restricted to the individually determined spasticity-free (active control) range (*Restricted-range*) with concomitant a-tDCS over the sensorimotor cortex of the affected hemisphere. Group 2 practiced non-restricted (whole range) reaching training (*Non-restricted-range*) with similar a-tDCS. Group 3 practiced reaching training in the Restricted-range condition as Group 1 but with sham stimulation at the same location. Outcomes were measured before (Pre), immediately after (Post), and 1 month after the 10-day intervention (Follow-up).

**Figure 2:**
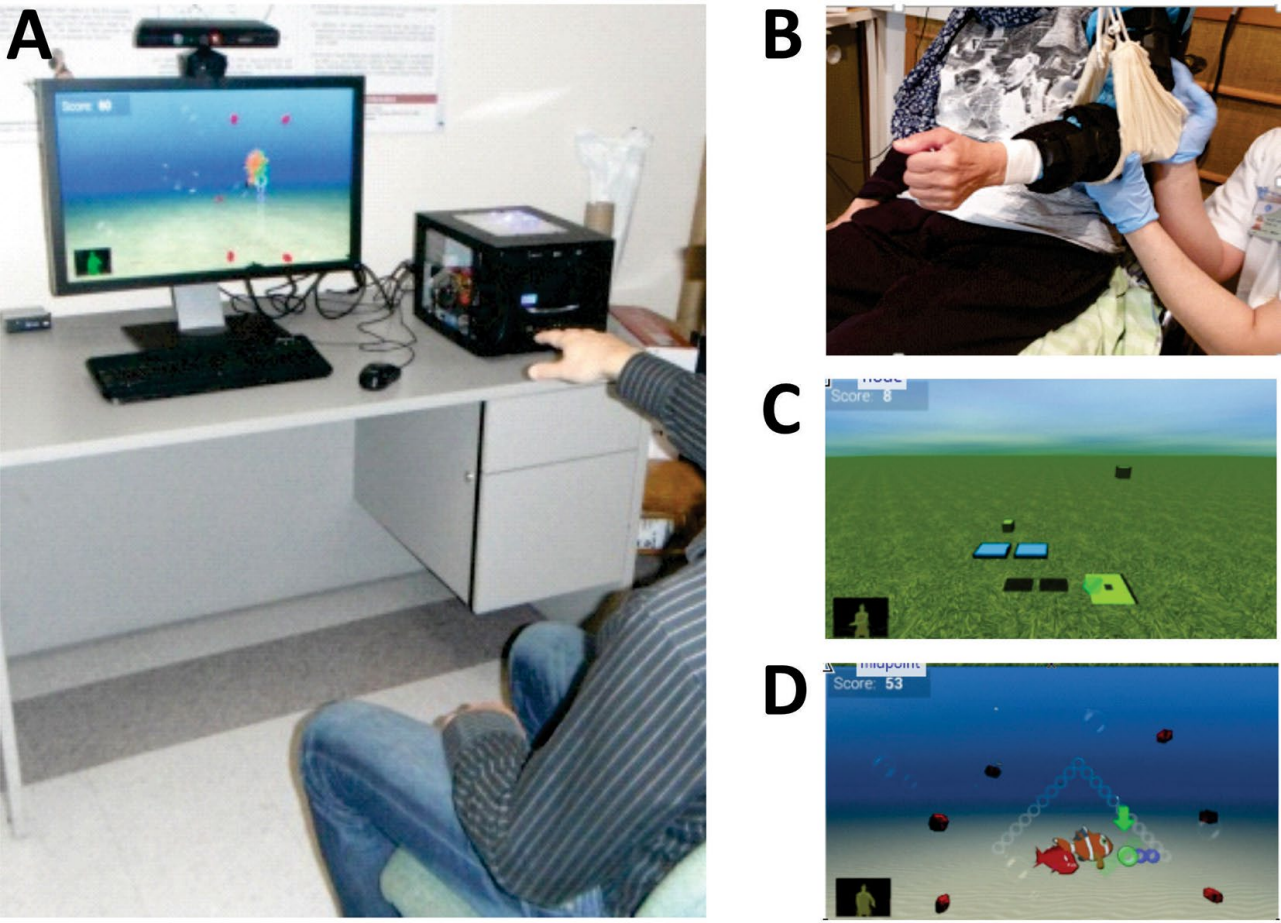
Illustration of virtual reality training environment. (**A**) Participant sat at 2 m distance from the screen and interacted with the game with one or both arms. (**B**) The arm was supported by a sling. Participants in the Restricted-range group wore a brace that limited their elbow extension to the previously determined elbow flexor tonic stretch reflex angle. Examples of the bimanual and unilateral arm activities are shown in C and D, respectively.

*Elbow range restriction*: Before training, elbow flexor TSRT was determined with the Montreal Spasticity Measure (MSM^21^) to define the spasticity-free elbow range in each participant (Fig. 1). The TSRT angle defined the limits of allowable elbow extension (i.e., the “restricted range”) during reaching. For the Restricted-range training group, UL movement was constrained using a brace (Breg Inc., Carlsbad, CA, USA) that blocked elbow extension beyond the TSRT angle. The Non-restricted-range training group wore the brace without imposing any elbow restrictions. For both groups, partial arm-weight support was provided via a sling system that assisted arm elevation during training and to avoid shoulder fatigue/pain without restricting arm movement (Fig. 2).

*Reaching training*: Participants sat on an armless chair, 2 m in front of a large screen, and practiced 3D reaching exercises delivered in VR. VR is an effective and motivating training environment^22^ used to standardize the training (activities, intensity, feedback) in all three countries. At trial inception, this was the recommended protocol to carry out the project while keeping costs to a minimum, as requested by the funding agencies (i.e., suitable methods and protocols for low-medium income countries). Each practice session lasted between 60 and 80 min and consisted of 50 min of active reaching training. Treatments were matched for duration and intensity across sites and guided by clinicians trained by the same study coordinator to ensure consistency of treatment delivery and progression^23^. The intervention was additional to conventional in- or outpatient therapy. Logbooks quantified participants’ received services outside the intervention (physiotherapy, occupational therapy, speech therapy, recreation, etc.).

Four VR activities engaged either the contralesional (unilateral) or both (bilateral) ULs (Fig. 2). Object distance was calibrated to lie within the active control zone (Restricted-range group) or within the patient’s effective arm length (Non-restricted-range group) before each session and progressed according to changes in this length. Thus, reaching distances may have been shorter in the Restricted-range group compared to the Non-restricted-range group with respect to elbow extension, while both groups used similar ranges of shoulder flexion and shoulder abduction/adduction. In VR, movements were tracked by a Kinect™ II camera (Microsoft Kinect V2, Redmont, WA, USA). Three unilateral activities required coordinated shoulder and elbow movements to reach different parts of the arm workspace: Controlled Movement, Lateral Reaching and Reaching Forward. The Controlled Movement game involved guiding the movement of a fish with one arm through different movement configurations on the screen (i.e., straight line, circle, triangle, figure of 8, square). Individuals were encouraged to guide their arm movement with the shoulder in ~ 90° flexion. The Lateral Reaching game involved moving tomatoes from a plant to a basket. The Reaching Forward game involved moving cups and cutlery from a counter to shelves and drawers. The Lateral Reaching and Reaching Forward games involved moving objects to the ipsilateral, contralateral, near, and far arm workspaces. The fourth activity required the coordination of both arms to catch falling apples and drop them into a container located laterally. Games were played in random order within each training session, according to patient preferences and therapist-determined training goals.

Difficulty levels were determined by increases in the number of items/objects, object placements, and playtime for each game^23^. Training progressed through 10 “difficulty levels” based on game progression guidelines, task success (reaching distance, speed, precision), clinical judgment, and patient preferences according to the ‘Challenge Point’ motor learning theory^24^. This theory suggests that learning is enhanced by optimally challenging the individual by manipulating task difficulty according to motor skill level and cognitive capacity.

Therapists provided similar encouragement and feedback during the sessions across centers. During familiarization trials, verbal feedback (knowledge of performance) about the required movements was given for each activity. For example, in the Reaching Forward game, feedback was provided regarding how the fork was placed in the tray. For the bilateral VR activity, feedback was provided about the use of the affected hand and compensations from the less-affected side. During gameplay, participants received positive feedback (movement quality, game score) and negative feedback about undesirable compensatory trunk movement (sagittal trunk displacement exceeding 5 cm). Total session/active training time (minutes), total number of movement repetitions, and success rate were extracted from game logs for each session. Exercise intensity was computed as the total number of movement repetitions/total active training time and expressed as repetitions/minute^23^.

*Non-invasive brain stimulation* (Soterix, New York, NY, USA) was applied for 30 min at the initiation of each training session. Two 5 × 7 cm saline-soaked surface electrodes were placed over sensorimotor areas of the affected hemisphere (anode: C3/C4 in the EEG 10–20 referencing system; cathode: contralateral supraorbital area). The stimulation level was increased from 0 to 1.5 mA in the first 30 s and decreased in the last 30 s of the stimulation train. Sham stimulation consisted of only the ramp-up and down stimulation, lasting for the first minute. Sham stimulation was used to mimic the a-tDCS intervention while not having any therapeutic effect so that the subject’s expectation of the effect of the intervention was matched between groups as closely as possible.

### Outcomes

Primary outcomes were the TSRT angle of elbow flexors and reach-to-grasp arm kinematics. Secondary outcomes were the velocity sensitivity of the TSRT (μ) and UL clinical measures at Body Structure and Function (motor impairment) and Activity levels of the International Classification of Function.

### Primary outcomes

1) *TSRT angle:* The elbow flexor (biceps brachii, short head, BB) TSRT angle was measured with the MSM, a portable device consisting of two electromyography (EMG) channels (Procomp 5, Thought Technology, Montreal, Canada), an electro-goniometer (P2200; Novotechnik, Southborough, MA, USA), and dedicated software. Using MSM, the TSRT measure has moderate-to-good intra- and inter-evaluator reliability for the measurement of post-stroke elbow spasticity (ICC = 0.68, 95% CI 0.19, 0.90) with a mean inter-evaluation difference of 5.9° (95% CI 3.7, 15.5), and MCD_95_ and MCD_90_ values of 32.4° and 27.2°, respectively^25^. For TSRT assessment, participants sat with their affected arm supported on a pillow, and their shoulder in ~ 45° flexion and ~ 30° abduction. After skin preparation, EMG electrodes (Ambu® Blue SensorP, Ballerup, Denmark) were placed over BB, short head, and triceps brachii – lateral head (TB) motor points. The electro-goniometer rotational axis was aligned with the elbow rotational axis and attached to the lateral arm and forearm. Participants performed a maximal voluntary elbow flexor contraction to adjust EMG gain. The MSM ensured that elbow flexor EMG had minimal initial activity and the elbow was maintained within ± 10° of the initial angle before each stretch^21^. The elbow was stretched using a bell-shaped velocity profile from full flexion (~ 50°) to full extension at slow, medium, and fast speeds, randomly specified by the software via a series of auditory tones. Participants were instructed to relax completely without assisting or resisting the displacement. At least 20 stretches were done with a minimum of 10 s between stretches to minimize fatigue and ensure recovery of muscle fibers from effects of the previous stretch^26^.

In the MSM software, raw data (EMG and goniometer) were synchronized and collected at 2048 samples/s. EMG signals were amplified at a gain of × 500 and further band-pass filtered (10–1000 Hz). After each stretch, MSM identified the angle and velocity at the time of EMG onset, called the dynamic stretch reflex threshold (DSRT^21^). The EMG onset was defined as the time when the EMG activity rose above 2xSD of the baseline activity for at least 25 ms. The software computed the linear regression line through the DSRTs on an elbow angular velocity/displacement plot, the velocity-sensitivity (μ), the square of the correlation coefficient (r^2^), and the x-axis intercept (i.e., TSRT angle). The x-axis intercept (when velocity equals zero) defined the TSRT angle, where a low angle corresponded to high spasticity. The “spasticity range” was determined from the TSRT angle to the full elbow extension angle (degrees).

2) *Kinematics of reach-to-grasp movements (Test-Task)*, requiring different amounts of elbow extension, were recorded at each time point (Pre, Post, Follow-up) with a room- and body-calibrated tracking electromagnetic system (G4, Polhemus, Colchester, VT, USA) at 120 Hz. Five magnetic transmitters were placed on the first metacarpophalangeal joint, proximal 1/3 of the dorsal forearm, the mid-lateral arm surface, mid-point of the acromial superior-lateral border, and mid-sternum. Participants sat in front of a table on an armless chair with their performing elbow in slight flexion (30°) and the arm alongside the body. At a verbal cue, participants reached to grasp a cone (6 cm base diameter, height ~ 18 cm) as fast and as precisely as possible, held or touched the cone (if they were unable to grasp it) for 2 s, lifted it and brought it towards the chin. A mid-sagittal (ego-centered) reference frame defined four arm-referenced target locations (Near Central, NC; Far Central, FC; Ipsilateral, IL: Contralateral, CL), whereas arm length was defined as the distance from the medial mid-axillary border to the distal wrist crease with the elbow extended. NC and FC targets were located at 2/3rd and at full arm’s length in the mid-sagittal plane, respectively. IL and CL targets were placed ~ 20 cm to the right and left of the FC target, respectively, for the right arm and opposite for the left arm. After two initial practice trials per target, participants repeated two sets of 40 trials (20 trials × 4 targets = 80 movements, randomized). Rest between sets and trials was provided to avoid fatigue.

Kinematics were analyzed for performance (endpoint) and movement pattern (joint/segment) variables^27^. Sensor data were filtered using a 3rd-order Butterworth low-pass filter with a 6 Hz cutoff and a zero lag (i.e., run twice in reverse order). Endpoint trajectory temporal variables were characterized by peak velocity (cm/s) and smoothness (zero crossings in the acceleration-time profile). Spatial trajectory variables analyzed were elbow extension, shoulder abduction/adduction, and shoulder flexion ranges, arm-plane angle path length (°), and forward trunk displacement (cm) as movement quality measures based on standard 3D kinematic reconstruction^28^. We also computed a model-based stochastic multivariate measure of performance (Hellinger’s distance, HD^29^), indicating the difference (i.e., distance) between the movement profile of each joint (elbow extension, shoulder abduction/adduction, shoulder flexion) and that of a group of healthy age-matched subjects previously collected^30^. Using a logit operator (Logit_HD), HD quantifies the distance between probability density functions and ranges between 0 and 1, where 0 represents an identical distribution^30,31^. The distance to the most similar healthy individual in the database was measured for each participant, joint, and target. The distribution parameters (means, covariance matrices, weights) were estimated using the expectation–maximization algorithm, where the number of components was found based on the Bayesian information criterion ^32^. We also computed the Kullback–Leibler (Log-KLDFP) divergence from control of each joint angle for each patient to quantify the divergence of the distribution representing the motion of a control individual from the distribution representing the patient’s movement^29,31^.

### Secondary outcomes

*3) Velocity sensitivity of TSRT (μ)* was computed by the MSM as the cotangent of the slope angle of the regression line (μ = − ctn(α), ms), where lower values denoted lower sensitivity.

*4) Clinical outcomes* were collected at the Body-Structure and Function Level for UL movement and coordination (66-pt), tactile sensation (12-pt), and passive range of motion (20-pt) with the valid and reliable Fugl-Meyer Assessment for the Upper Extremity (FMA-UE^33^). Changes in FMA-UE scores are thought to indicate recovery of elementary movements^5^. Resistance to passive stretching of elbow flexors and extensors was measured with the MAS^18^ on a scale of 0 to 4-pt. MAS has poor-to-good inter-rater reliability^34^.

In addition, UL movement at the Activity Level was assessed with the Functional Activity Score (FAS) of the 6-item Streamlined Wolf Motor Function Test (S-WMFT^35^). Mean scores were reported. The S-WMFT has good concurrent (Spearman ρ = 0.69) and predictive validity (Spearman ρ = 0.68) with FMA, and a mean standard response of 0.41^36^. Changes in S-WMFT reflect improvement through recovery and compensation^5^.

### Data management

Evaluation and testing procedures were standardized via written guidelines, videos, and team meetings to ensure inter-site consistency. The oversight committee comprised the co-PIs (MFL, DGL) and an uninvolved individual. The data monitoring and management committee (SFT, JS, MCB), led by MCB ensured the uploading of coded data to a secured repository. Adverse events were reported to local ethics committees, and mitigating procedures were followed.

### Statistical analyses

The sample size was based on preliminary data showing an average elbow-flexor TSRT change of 10° in stroke patients with spasticity who underwent a 2-week UL intervention, compared to 2° in a control group (unpublished data). Considering an α-level of 5% and a 95% power (effect size = 2.23) to detect differences using a mixed-design ANOVA (G*Power 3.1.1), the minimal sample size was 13 patients/group. For the initial analysis, the mixed model analysis included one between-subjects factor – Group with three levels (personalized training + a-tDCS, non-personalized training + a-tDCS and personalized training + sham-tDCS), and one within-subject factor – Time with three levels (Pre, Post and Follow-up for raw data; Post–Pre and Follow-up-Pre). One-way ANOVAs were used to evaluate change scores, noting confidence intervals (CIs). Between-group differences in proportions were analyzed with Chi-Square tests. Initial *p* values were 0.05 corrected for multiple comparisons using Bonferroni corrections. Effect sizes (ES) were computed from ANOVAs or Kendall’s W (for Friedman).

For the secondary statistical analysis, subjects were divided according to 2 intervention factors: 1) Restricted-range, and 2) Non-restricted range. Normal distributions were verified for each measure and group using Shapiro–Wilk normality tests and normal Q-Q plots. When normality criteria were unmet, data were normalized using reciprocal log or logit transformation functions.

Descriptive statistics highlighted the main demographic and clinical participant characteristics. Statistical analysis was performed using Linear Mixed Models (LMM), in which the linear predictor contains random and fixed effects. The fixed effects for all measures were: training range or “Range” (Restricted, Non-restricted) and “Time” (Pre, Post, Follow-up), and their interaction. In addition, kinematic and stochastic measures for each joint included a fixed effect of “Target” (NC, FC, IL, CL) and the interaction of Target with the other fixed factors. The random effect was “Subject”. Each measure was first analyzed using the appropriate full model, and then backward elimination was used to remove non-significant factors (i.e., factors with a low contribution to the covariance matrix), where *p* < 0.05 was used for inclusion. At each step, we considered excluding one of the interactions or one of the main effects unrelated to any active interaction. Overall, the effect size of each final model was assessed by conditional R^2^ ^37^. Post-hoc analysis was conducted with Holm’s correction (i.e., sequential Bonferroni) for multiplicity to maintain a ‘global alpha’ < 0.05.

## Results

3106 patients were screened and 52 randomized to one of 3 treatment groups (Supplementary Fig. 1). Since this study is a proof-of-concept study rather than a full RCT, the sample size is small. Time limitations and cost constraints did not allow us to extend the sample size, as done for full RCTs such as classical studies of Constraint-Induced Movement Therapy^38,39^. Forty-nine patients (20 females; 24 right-sided/dominant hemiparesis, aged 53.5 ± 11.7 yr and 70.8 ± 43.5 days since stroke) started the trial, but 3 dropped out before completion resulting in a final total of 46 patients. In the initial distribution, there were 16 participants in Group 1, 15 in Groups 2, 3 (Table 1). Numbers were balanced across sites. Baseline characteristics and clinical data of the original three groups can be found in Table 1. Of these, 8 had missing PRE training kinematic data, and 3 had missing Post and Follow-up data. Thus, a total of 35 patients were included in the secondary analyses. When grouped by treatment modality, 25 patients trained in the Restricted-range and 10 patients trained in the Non-restricted-range (Table 1).

The initial overall mean FMA-UE score was 32.6 ± 13.1 pt (range 14–57 pt), with 43% (n = 15) having severe (FMA = 0–29 pt), 43% (n = 15) having moderate (FMA = 30–49 pt) and 14% (n = 5) having mild impairment (FMA ≥ 50 pt^40^). All groups had similar reaching training (VR) time and intensity with an average overall time in active training of 452 ± 78 min for 10 sessions and a per-session training time of 48 ± 5 min corresponding to 7.1 ± 2.5 repetitions/min^23^ (Table 1). The time engaged in other therapies was also similar between groups.

### Initial analysis

TSRT angle increased in all groups from Pre (101.0 ± 22.3°) to Post (109.5 ± 25.9°) and was maintained at Follow-up (109.5 ± 30.0°; F_2,86_ = 2.994, *p* = 0.05, ES = 0.570) with no change in μ (Supplementary Table 1). There was a significant group effect with both Groups 1 and 3 changing more than Group 2 (F_2,43_ = 5.107, *p* = 0.01, ES = 0.192). However, effects were not significant when change scores were compared and adjusted for initial baseline differences. All groups improved in FMA-UE scores (z = 8.233–19.404, *p* = 0.000–0.016; ES = 0.257–0.647; Supplementary Table 1) with no group differences. Overall medians were 33.5 (IQR 23.0) pt at Pre-test, 41.5 (IQR 22.0) pt at Post-test and 44.0 (IQR 45) pt at Follow-up. At Post-test, average increases were 4.8pt for Group 1, 4.7 pt for Group 2 and 6.8 pt for Group 3 (F_1,43_ = 9.510, *p* = 0.004, ES = 0.181). FMA scores continued to improve at Follow-up with increases from Pre-test of 5.7, 6.7 and 8.8pt, respectively. FMA improvements exceeded the MCID of 5.25 pt^41^ only in Group 3 at Post-test but for all groups at Follow-up.

### Secondary analysis

#### Primary outcomes

**Figure 3:**
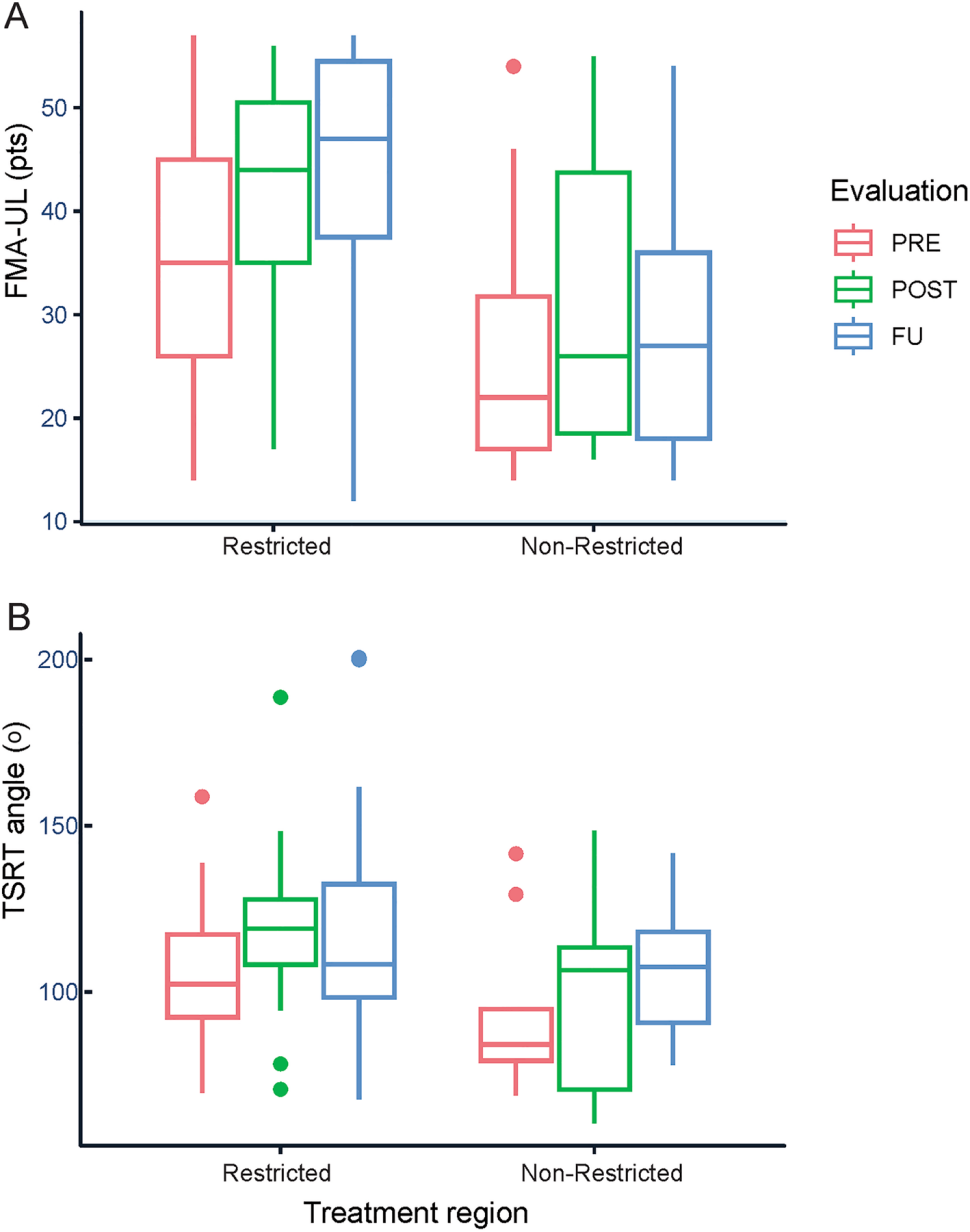
Results of changes in Fugl-Meyer Assessment of the Upper Limb (FMA-UL) and the tonic stretch reflex (TSRT) angle for all subjects in both training groups at Pre, Post and Follow-up assessments.

**Figure 4:**
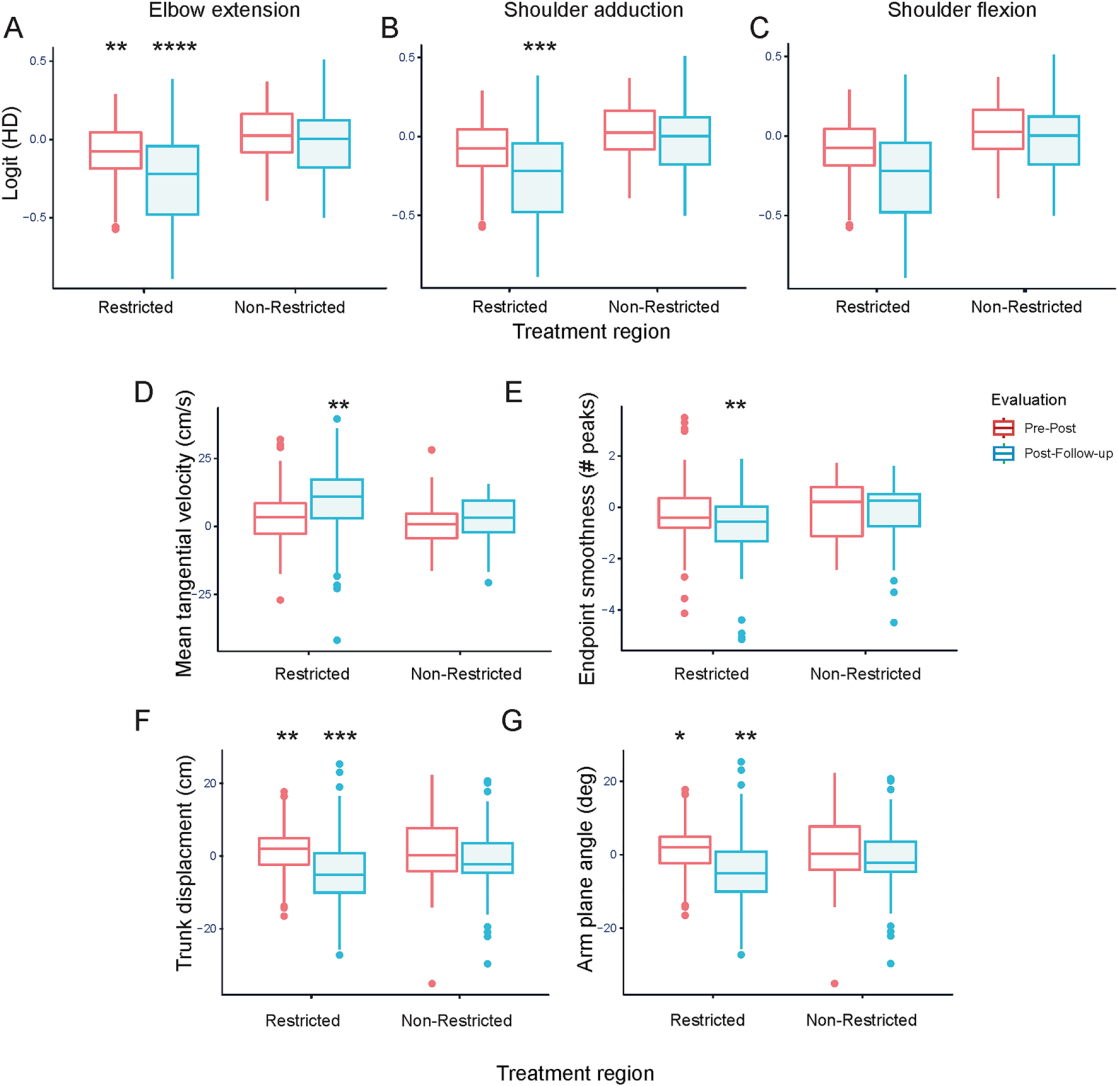
Results of changes in key kinematic outcomes from Pre to Post (red) and from Post to Follow-up (blue) in the two training groups for all four targets. (**A**–**C**): joint kinematics – Hellinger’s distance (HD) values indicating the difference (i.e., distance) between the movement profile of each joint (elbow extension, shoulder adduction, shoulder flexion) and that of a group of healthy age-matched subjects; (**D**,**E**): endpoint velocity and smoothness; F: trunk displacement; G: arm-plane angle. Box plots represent the interquartile range (IQR), the middle line of the box represents the median, whiskers represent the variability outside the upper and lower quartiles, and dots represent outliers (values above Q3 plus 1.5 times IQR or below Q1 minus 1.5 times IQR). Significant interaction effects are illustrated by asterisks (* *p* < 0.05; ** *p* < 0.01; *** *p* < 0.001; **** *p* < 0.0001).

1. Intervention effects on TSRT angle
  Changes in TSRT angles were explained by Time (R^2^ = 0.41, Table 2). TSRT angles increased in both groups from Pre to Post (t_64.0_ = 2.1, *p* < 0.05), and the improvement was maintained at Follow-up (t_64.4_ = 2.6, *p* < 0.05) with no differences between Post and Follow-up. For clarity, values of FMA-UE and TSRT angles are illustrated for each group and time-point in Fig. 3.
2. Intervention effects on arm kinematics
  40% of subjects were unable to grasp and lift the cone, equally distributed between training groups. However, this did not affect the results since only the arm endpoint and joint kinematics were included in the analysis. Pre-, Post- and Follow-up scores of clinical tests and kinematic outcomes for the Contralateral target for each group of subjects in the original grouping can be found in Supplementary Table 1.
  a) *Endpoint tangential velocity*: Changes were explained by Target, Time, and the interaction between Time and Range (R^2^ = 0.85, Table 2). Tangential velocity values for the NC and IL targets were similar and movements to these two targets were slower than for FC (FC vs. NC t_358_ = 5.5, FCvsIL t_358_ = 5.6, *p* < 0.001) and CL (CLvsNC t_358_ = 8.1, CLvsIL t_358_ = 8.2, *p* < 0.001) targets. Movements to CL were faster than to FC (t_358_ = 2.6, *p* < 0.05). Movements at baseline were slower in patients with stroke compared to previous data from age-matched healthy subjects performing the same tasks^31^ (see Table 3). The improvement (faster movements) from Pre to Follow-up with Restricted-Range training was significantly greater than that obtained with Non-Restricted-Range training (t_358.6_ = 2.7, *p* < 0.01), while there were no differences in the effects of Range at Post compared to Pre. Overall effects for each kinematic variable for all targets are shown in Fig. 4.
  b) *Movement smoothness (acceleration zero crossings)*: Changes in values were explained by Time, and Time-by-Range interaction (R^2^ = 0.81, Table 2). Endpoint movements were more fragmented in patients with stroke compared to controls at baseline (Table 3). The improvement (fewer zero crossings) from Pre to Follow-up with Restricted-range training was significantly larger than that with Non-restricted-range training (t_361.8_ = − 3.0, *p* < 0.01), without differences between Pre and Post (Fig. 3).
  c) *Trunk displacement:* Values were not transformed since they could not be normalized with a standard transformation. However, since the F-test is robust even under normal distribution violations, we pursued the analysis^42^. Changes in trunk displacement values were explained by Target, Time, and Time-by-Range interaction (R^2^ = 0.85, Table 3). Trunk displacement was greater at baseline for patients with stroke compared to controls. Trunk displacement when reaching for IL and FC targets was similar and greater than for NC (NCvsIL t_358_ = − 10.0, NCvsFC t_358_ = − 9.8, *p* < 0.0001) and CL (CLvsIL t_358_ = − 4.5, CLvsFC t_358_ = − 4.3, *p* < 0.0001) targets. Trunk displacement for NC was less than CL (t_358_ = − 5.5, *p* < 0.001). Trunk displacement was initially smaller for the Restricted-range group and the reduction in trunk displacement following training was larger for the Non-restricted-range compared to the Restricted-range group between Pre and Post (t_358.1_ = 2.1, *p* < 0.01) and Pre and Follow-up (t_358.4_ = 3.2, *p* < 0.001).
  d) *Arm-plane angle path length*: Changes in values were explained by Target, Range, Time, and Time-by-Range with R^2^ = 0.86 (Table 2). Path length was greater at baseline for patients with stroke compared to controls (Table 3). Path length towards NC was smaller than towards all other targets (FC t_358.0_ = − 9.8, *p* < 0.001; IL t_358.0_ = − 10.0, *p* < 0.001; CL t_358.0_ = − 5.5, *p* < 0.001). Path length towards CL was higher than towards FC (t_358.0_ = 4.3, *p* < 0.001) and IL (t_358.0_ = 4.5, *p* < 0.001). The decrease in path length between Pre and Post was larger when training in the Restricted compared to the Non-restricted-range (t_358.1_ = − 2.1, *p* < 0.05), and this difference increased at Follow-up (t_358.4_ = − 3.2, *p* < 0.01; Fig. 3).
  e) *Stochastic measures of joint movement patterns, Hellinger’s distance (HD)*:
  *HD-elbow-extension*: Elbow extension ranges for each target and at each timepoint are shown in Supplementary Table 2. Changes in values were explained by Target, Time, and Time-by-Range interaction (R^2^ = 0.85, Table 2). HD-elbow for CL was higher than for FC (t_358_ = 3.6, *p* < 0.01) and IL (t_358_ = 2.9, *p* < 0.05). HD values for other targets were similar. The decrease in HD between Pre and Follow-up was larger for Restricted compared to Non-restricted-range training (t_358.1_ = − 2.7, *p* < 0.01), and this difference increased at Follow-up (t_358.7_ = − 5.1, *p* < 0.0001; Fig. 3).
  *HD-shoulder-adduction*: Changes in HD-shoulder-adduction values were explained by Target, Range, Time, and Time-by-Range interaction, with a goodness-of-fit R^2^ = 0.81 (Table 2). HD values for CL were higher than for IL (t_358.0_ = 13.9, *p* < 0.001). HD values between other targets were similar. The decrease in HD between Pre and Follow-up was larger when training in the Restricted compared to the Non-restricted-range (t_359.0_ = − 4.7, *p* < 0.001), with no differences between Pre and Post (Fig. 3).
  *HD-shoulder-flexion*: Changes were explained by Time with R^2^ = 0.71 (Table 3; Fig. 3).

**Table 2:** Mean (SD) values for Pre-, Post- and Follow-up clinical tests scores and kinematic outcomes of reaches to the contra-lateral target for each intervention (“Range” [Restricted N = 25, versus Non-restricted N = 10]).

	Measurement time	Pre		Post		Follow-up	
	Intervention factor	Restricted range	Non-restricted range	Restricted range	Non-restricted range	Restricted range	Non-restricted range
Clinical Measures	TSRT (°) ***	105.1 (21.0)	92.9 (24.0)	119.2 (24.2)	97.8 (29.5)	119.3 (36.1)	107.9 (20.3)
	µ (s)	0.088 (0.082)	0.066 (0.091)	0.155 (0.200)	0.061 (0.052)	0.094 (0.084)	0.079 (0.064)
	FMA (pt) ***	34.9 (12.3)	26.7 (13.7)	41.2 (11.4)	30.7 (15.1)	43.6 (12.6)	29.8 (14.5)
	WMFT -FAS (pt) ***	2.84 (0.94)	2.22 (1.19)	3.30 (0.96)	2.4 (1.2)	3.44 (0.90)	2.46 (1.27)
Kinematic measures, contra-lateral target	Mean wrist tangential velocity (cm/sec) ***; H = 32.79 (6.99)	***45.45 (19.60)***	***45.55 (15.89)***	***49.18 (18.91)***	***46.89 (16.66)***	***57.48 (19.10)***	***48.45 (14.13)***
	Acceleration zero crossings (#) ***; H = 1.45 (0.41)	***3.51 (1.55)***	***4.73 (2.17)***	***3.30 (1.48)***	***4.30 (1.63)***	***2.81 (1.28)***	***4.12 (1.63)***
	Trunk forward displacement (cm) ***; H = 1.05 (0.49)	***4.93 (5.09)***	***9.26 (7.66)***	***5.18 (5.80)***	***8.29 (7.04)***	***4.23 (5.84)***	***7.09 (6.36)***
	Arm plane angular path length (deg) ***; H = 6.25 (20.53)	***32.51 (10.20)***	***41.13 (19.80)***	***33.95 (11.44)***	***42.17 (18.36)***	***29.22 (13.96)***	***37.88 (19.50)***
	Logit HD elbow extension ***	***0.57 (0.29)***	***0.69 (0.32)***	***0.52 (0.32)***	***0.70 (0.29)***	***0.34 (0.35)***	***0.71 (0.33)***
	Logit HD shoulder adduction	***0.70 (0.28)***	***0.86 (0.34))***	***0.65 (0.28)***	***0.85 (0.33)***	***0.42 (0.40)***	***0.87 (0.34)***
	Logit HD shoulder flexion ***	***0.54 (0.25)***	***0.61 (0.29)***	***0.51 (0.26)***	***0.60 (0.32)***	***0.37 (0.32)***	***0.37 (0.32)***

*The random effect Center was retained in the final model. TSRT, Tonic Stretch Reflex Threshold; µ, velocity sensitivity of the stretch reflex; FMA, Fugl-Meyer Assessment; WMFT-FAS, Wolf Motor Function Test-Functional Activity Score; HD, Hellinger’s distance.

For the kinematic outcomes, values in a healthy age-matched group (H) performing the same tests and Hellinger’s distance (HD) from the most similar healthy control are reported in column 2. Significant differences between “Time” of measurement levels (Pre, Post, Follow-up) are also indicated in column 2 (***p < 0.001), while significant interactions between each “range” and “time” are emphasized in bold italics***.***

**Table 3:** Effects in the final model for each measure.

	Measure	T-n	Time	Range	Target	Time*range	R_c_^2^
Clinical Measures	tsrt (°)	Log	F_2, 64.5_ = 3.9, *p* < 0.05	–		–	0.41
	μ (s)*	Log	–	F_1, 97.8_ = 4.7, *p* < 0.05		–	0.20
	fma (pt)	None	F_2, 63.2_ = 28.2, *p* < 0.001	F_1, 33.0_ = 5.0, *p* < 0.05		–	0.91
	wmft fas (s)	None	F_2, 63.0_ = 19.6, *p* < 0.001	–	–		0.90
Kinematic measures, contra-lateral target	Mean wrist tang. velocity (cm/sec)	Log	F_2, 358.4_ = 23.2, *p* < 0.001	–	F_3, 358.0_ = 33.5, *p* < 0.001	F_2, 358.4_ = 3.7, *p* < 0.05	0.85
	Acceleration zero crossings (#)	Log	F_2, 361.6_ = 14.1, *p* < 0.001	–	-	F_2, 361.6_ = 4.6, *p* < 0.05	0.81
	Trunk forward displacement (cm)	None	F_2, 358.4_ = 13.6, *p* < 0.001	–	F_3, 358.0_ = 44.2, *p* < 0.001	F_2, 358.4_ = 5.2, *p* < 0.01	0.86
	Arm Plane Angle path length (deg)	Log	F_2, 358.4_ = 13.6, *p* < 0.001	–	F_3, 358.0_ = 44.2, *p* < 0.001	F_2, 358.4_ = 5.2, *p* < 0.01	0.86
	Logit HD Elbow Extension	Logit	F_2, 358.6_ = 19.9, *p* < 0.001	–	F_3, 358.0_ = 5.5, *p* < 0.01	F_2, 358.6_ = 12.8, *p* < 0.001	0.85
	Logit HD Shoulder Adduction	Logit	F_2, 358.9_ = 7.8, *p* < 0.001	F_1, 33.0_ = 7.0, *p* < 0.05	F_3, 358.0_ = 101.3, *p* < 0.001	F_2, 358.9_ = 11.2, *p* < 0.001	0.77
	Logit HD Shoulder Flexion	Logit	F_2, 364.0_ = 43.7, *p* < 0.001	–	–	–	0.71

*The random effect Center was retained in the final model. TSRT, Tonic Stretch Reflex Threshold; µ, velocity sensitivity of the stretch reflex; FMA, Fugl-Meyer Assessment; WMFT-FAS, Wolf Motor Function Test-Functional Activity Score; HD, Hellinger’s distance.

The initial model was measured ~ (range * time of measurement * target + (1|subject). The final model was found using a backward elimination technique. F and *p* values are presented for the significant factors in the final model for each measure. Effects and interactions that were not significant in any of the models are not presented in the table. Transformations (T-n) for normalizing the data were carried out before the analysis. R_c_^2^ is the conditional R^2^.

### Secondary outcomes

1. *Velocity sensitivity (μ)*
  There were no overall effects of Time while there was an effect of Range (R^2^ = 0.20, Table 3). Velocity sensitivity was higher for Restricted-Range compared to the Non-Restricted-Range training group (t_97.8_ = 2.2, *p* < 0.05).
2. *Clinical measures*
  *FMA-UE*: Changes were explained by Time and Range (R^2^ = 0.91, Table 2). FMA-UE scores increased from Pre to Post (t_63.1_ = 5.71, *p* < 0.001), with improvement maintained at Follow-up (t_63.1_ = 7.01, *p* < 0.001). Although there was a significant difference between the Restricted and Non-restricted-range training groups at PRE (t_32.98_ = − 2.23, *p* < 0.05), there were no interactions, suggesting that between-group differences were similar throughout the study. However, FMA-UE improvements exceeded the MCID (5.25 points^41^) only for the Restricted-range group at Post, and at Follow-up (Table 2). There were no intervention effects on MAS.
  *WMFT-FAS scores*: The change in WMFT-FAS was explained by Time, with R^2^ = 0.90 (Table 2). WMFT-FAS increased for both groups from Pre to Post (t_63.0_ = 4.6, *p* < 0.001), and the increases were maintained at Follow-up (t_63.4_ = 5.8 *p* < 0.001; Table 3).

## Discussion

We investigated the effects of a novel theory-driven approach on reaching training of the hemiparetic UL in patients with subacute stroke. Training was provided based on the individual’s specific elbow extension range impairment. Using TSRT assessment, in each patient we identified the range of his/her elbow movement in which disordered control of stretch reflex activation occurred in the form of elbow flexor spasticity. Repeated reaching training was conducted with the intentional restriction of elbow extension to the spasticity-free part of the range. We expected that this type of individualized practice would result in better arm movement quality, given the relative preservation of the ability to produce typical muscle activation patterns in that range^9^.

Clinical measures of UL impairment and activity limitation improved after the 2-week training period in both the Restricted- and Non-restricted range groups. While some of this improvement may have been due to spontaneous recovery^43^, the amount of recovery would have been balanced between groups due to patient randomization. In a previous study, equivalent dose task-oriented training of a similar time frame and stroke cohort did not improve UL outcomes more than standard therapy^44^. This study showed that there was no advantage to providing more than twice the mean therapy dose (mean, 27 h) compared with the average 11 h received by the usual care group, showing that substantially more therapy time was not associated with additional motor improvement. However, in this proof-of-concept study, training UL reaching in the personalized spasticity-free elbow range over a 2-week period was sufficient to demonstrate an improved arm function together with a demonstrable increment in movement quality: faster and smoother reaches, smaller arm-plane path length (less shoulder compensation^45^, and closer-to-normal elbow and shoulder movement patterns (Hellinger’s distance), without an increase in compensatory trunk movement (Table 2). Although training reaching movement in an unrestricted elbow range of motion also improved overall UL function, it did not improve arm kinematics. This implies that some of the overall functional improvements assessed clinically may have been accomplished by the involvement of motor compensations. Although some clinical tests may be sensitive to such compensatory effects, monitoring of recovery by restitution of elementary constituents of healthy-typical movement patterns requires the analysis of arm kinematic variables^46^.

### Effects of training in VR environment

The personalized training program was implemented under conditions assumed to enhance neuroplastic changes. The overall improvement in clinical scores in both groups may be due to the use of the VR platform, which provided high-repetition practice, feedback, and gradual increases in difficulty levels. VR training interventions were shown to improve UL impairment and activity scores without accounting for improvements in kinematics^47^. Thus, overall improvements may partly be due to the increased practice intensity^48^ afforded by the motivating VR interface^49^.

### Effects of training in a restricted range

The results do not unequivocally support the hypothesis that training in the restricted range would increase the elbow flexor TSRT since similar improvements in elbow TSRT angles and clinical outcomes occurred in both groups. This suggests that repetitive UL training had an overall positive effect on increasing the active elbow extension range. However, only when training was specifically structured to avoid evoking ‘abnormal’ movement patterns in the restricted-range training group did participants produce movements that were more like those made by healthy subjects.

We implemented personalized impairment-based reaching training defined according to the individual’s active range of elbow extension (i.e., defined by the TSRT angle), which was associated with their specific elbow motor impairment. By identifying the angle at which elbow flexor spasticity began to interfere with elbow extension, we could shape the training so that repetitive reaching could be done without evoking unwanted elbow flexor muscle resistance leading to abnormal movement patterns^31^, and potentially, to greater motor compensations due to maladaptive plasticity^50^. Although we did not record agonist and antagonist EMG activity in the current study, previous studies have demonstrated that reciprocal muscle activation patterns are preserved when movements are restricted to the active elbow control range^9^ and normal error correction strategies^6^ could be used when movements were made within the active elbow range compared to those made in the spasticity range. We implemented these findings by limiting elbow movement to the active range so that patients could use good quality movement patterns when practicing the diverse reaching tasks in VR. Only a few studies have reported improvements in reaching kinematics following training in VR environments when feedback on specific movement elements was provided^49,51^.

Participants who trained in the Restricted-range showed an overall improvement in UL movement compared to the Non-restricted-range training group. However, the movement quality improvements were insufficient to substantially increase clinical motor impairment and activity scores. It is likely, however, that increases in the active range of one joint (elbow) may have been too modest to lead to meaningful changes in overall UL function and that future trials could shape training according to multiple joint limitations. Another explanation is that clinical scales are not sensitive enough to the specific effects of training on movement-quality variables that are detected by kinematic analyses^46^. Training-related improvement in clinical scores reflects not only true recovery by restitution but may also reflect a functional benefit accomplished using compensatory movement patterns. Our findings support a personalized impairment-based training approach based on the restriction of elbow joint motion during training to the spasticity-free angular range for better outcomes that could be considered in future clinical trials.

### Feasibility of combining technologies

Combining technologies in a theoretically driven treatment intervention was successfully implemented across all centers. Aside from equipment cost, common barriers to technology uptake (i.e., lack of knowledge, education, awareness, and access^52^) were overcome by providing clinicians with custom-written manuals (e.g., MSM, Jintronix system), evaluation protocols, and guidelines. Documentation was supplemented by virtual and in-person training sessions, regular team meetings, and problem-solving opportunities with peers and specialized technicians when needed.

### Trial limitations, potential sources of bias

The decision to limit the analysis to the effects of the Restricted vs Non-Restricted elbow range in a secondary analysis was based on our preliminary finding that there was no difference in the effects of training with tDCS. The lack of effect is consistent with more recent results that non-invasive brain stimulation such as tDCS and rTMS only showed main effects on training outcomes when patients were stratified according to the availability of functional reserve in the corticospinal system of the lesioned hemisphere^53^. We did not evaluate corticospinal integrity, and therefore, this stratification was not done. The lack of stratification combined with the 3-arm design and the small number of subjects likely contributed to the inconclusive results of the effect of tDCS on reaching ability in our subjects.

Our results provide data as a proof-of-concept for the use of personalized impairment-based training restricted to the patient’s active elbow control zone. The small sample size may have affected the robustness of our results. Although we had good adherence (88%), some data were lost primarily due to pre-post scheduling (e.g., bringing patients back to hospital centers for the post-treatment testing session) or technical issues (e.g., connectivity during online VR sessions). For example, since the software was meant to be run on an online platform, if there were wifi connectivity problems, we arranged for the VR software to be run as a stand-alone platform on the local computer. Since the VR technology was not accessible in one country (India), the technology was provided by the research project. In addition, the small number of subjects was partly due to the difficulty in recording kinematics in different clinical settings. For example, some data were lost or unusable because of errors in the orientation of recording electrodes. Bias was minimized by blinding personnel responsible for randomization, group allocation, clinical and kinematic evaluation, treatment, and data analysis. Also, all participants were trained while wearing the restrictive elbow brace and arm-sling. The actual reaching range during training for each group was not recorded although the VR program randomly assigned target locations in each task according to the arm workspace calibrated to the individuals’ reaching distance. This was full elbow extension for the Non-restricted group and elbow extension limited by the brace to the elbow flexor TSRT angle in the Restricted group. However, the lack of this information is a limitation of the study.

The current study results cannot be generalized to other outcomes, such as health-related quality of life and community participation. Neither can they be generalized to other stroke sub-groups. Finally, results should be interpreted considering that patients were also receiving standard care in the three different countries and, although this was monitored and found to be equivalent between training groups (Table 1,^23^), there was no control over the exact intensity of the standard treatment.

### Conclusions and recommendations

Our study showed that short-term personalized impairment-based training of UL reaching led to improved clinical scores along with better movement quality compared to non-personalized training. These results suggest a potential direction based on individualized impairment-based interventions for future studies aimed at maximizing UL recovery in individuals with sub-acute spastic hemiparesis. Benefits could have been more substantial with more intensive training and impairment-based training based on range limitations in other UL joints. However, the benefit of augmented therapy is reportedly small^54^ which is likely related to the generalized nature of therapeutic practice^5^, which does not consider the specific motor impairment of the patient from a theoretical motor control perspective. To move beyond the limitations of current interventions, resolving the individual’s specific motor impairment deserves more attention.

### Supplementary Information

Supplementary Figure 1.

Supplementary Tables.

## Data Availability

The datasets generated and/or analyzed during the current study will not be publicly available due to patient confidentiality rules, but anonymized data is available from the corresponding authors upon reasonable request. Raw clinical and kinematic data for all subjects can be accessed at^55^.
