# Supplementary figures and images for: ENHANCE proof-of-concept three-arm randomized trial: effects of reaching training of the hemiparetic upper limb restricted to the spasticity-free elbow range
Source: Sci Rep. 2023 Dec 22;13:22934. doi: 10.1038/s41598-023-49974-6 (PMC10739929; doi:10.1038/s41598-023-49974-6)

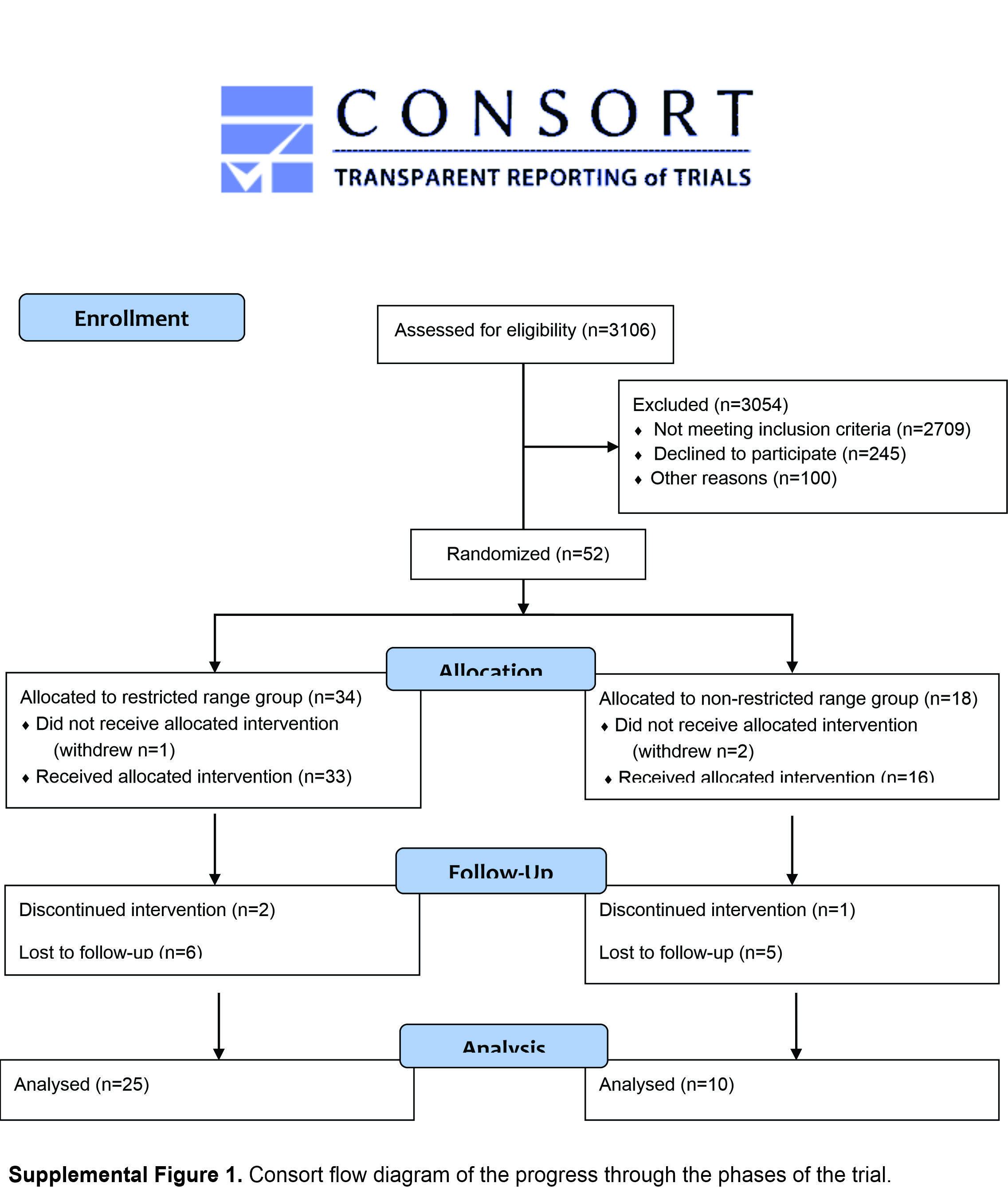

Supplement: Supplementary file 1 — Supplementary Figure 1. [file 41598_2023_49974_MOESM1_ESM.jpg]
