# Supplementary material for: ENHANCE proof-of-concept three-arm randomized trial: effects of reaching training of the hemiparetic upper limb restricted to the spasticity-free elbow range
Source: Sci Rep. 2023 Dec 22;13:22934. doi: 10.1038/s41598-023-49974-6 (PMC10739929; doi:10.1038/s41598-023-49974-6)
Supplement: Supplementary file 2 — Supplementary Tables. [file 41598_2023_49974_MOESM2_ESM.docx]

**ENHANCE proof-of-concept trial: Effects of reaching training of the hemiparetic upper limb restricted to the spasticity-free elbow range**

**Authors:** Mindy F. Levin PhD, Sigal Berman, PhD, Neta Weiss MSc, Yisrael Parmet PhD, Melanie C. Baniña, PhD, Silvi Frenkel-Toledo, PhD, Nachum Soroker, MD, John M. Solomon, PhD, Dario G. Liebermann, PhD

**Table S1.** Pre-, Post- and Follow-up scores of clinical tests and kinematic outcomes for the Contralateral target for each group of subjects. For kinematic outcomes, mean (SD) scores in a healthy age-equivalent group (H) performing the same test are indicated in the first column. Values are mean (SD) except where indicated as median (IQR, interquartile range).

*= Repeated Measures ANOVA; Ϯ = Friedman’s ANOVA by group; ** between group effect (Group 1 and Group 3 are different from Group 2)

|  | Group 1 (n=16) |  |  | Group 2 (n=15) |  |  | Group 3 (n=15) |  |  |
| --- | --- | --- | --- | --- | --- | --- | --- | --- | --- |
| **Primary Outcomes** | **Pre-test** | **Post-test** | **Follow-up** | **Pre-test** | **Post-test** | **Follow-up** | **Pre-test** | **Post-test** | **Follow-up** |
| **TSRT (°)*** | 113·2 (21·2) | 117·9 (24·9) | 117·1** (28·2) | 88·4 (21·8) | 93·3 (26·7) | 100·9** (28·5) | 100·7 (17·4) | 116·7 (19·1) | 110·0** (32·9) |
| **Spasticity range* (°)** | 66·8 (21·2) | 62·1 (24·9) | 63·5** (26·8) | 91·6 (21·8) | 86·7 (26·7) | 79·1** (28·5) | 79·3 (17·4) | 63·3 (19·1) | 71·4 (29·0)** |
| **FMA (66)**  **median (IQR)** | 37·5 (21·0) | 45·0 (20·0) | 49·0 (28·0)Ϯ | 25·0 (21·0) | 28·0 (32·0) | 32·0 (33·0) Ϯ | 33·0 (13·0) | 41·0 (35·0) | 43·0 (12·0) Ϯ |
| **Secondary Outcomes** |  |  |  |  |  |  |  |  |  |
| **WMFT – FAS*** | 2·80 (1·01) | 3·35 (1·08) | 3·32 (1·18) | 2·34 (1·24) | 2·54 (1·19) | 2·90 (1·31) | 2·76 (0·69) | 2·98 (0·84) | 3·21 (0·69) |
| **WMFT**  **median score** | 28·76 (43·14) | 17·38 (34·22) | 24·55 (42·52)Ϯ | 42·63 (42·96) | 32·24 (37·90) | 26·01 (38·26)Ϯ | 22·85 (26·79) | 20·63 (27·32) | 12·25 (20·97)Ϯ |
|  |  |  |  |  |  |  |  |  |  |
| **Mu (s)**  **median (IQR)** | 0·061 (0·110) | 0·082 (0·100) | 0·120 (0·050) | 0·030 (0·030) | 0·024 (0·090) | 0·025 (0·060) | 0·046 (0·060) | 0·095 (0·140) | 0·061 (0·230) |
| **Endpoint movement time* (s)** H=0·705 (0·157) | 1·734 (0·632) | 1·676 (0·579) | 1·477 (0·631) | 2·055 (0·829) | 2·147 (0·811) | 1·876 (0·658) | 1·840 (0·482) | 1.751 (0·516) | 1·434 (0·455) |
| **No. peaks*** H=1·45 (0·41) | 3·64 (1·80) | 3·23 (1·47) | 2·99 (1·58) | 4·74 (2·30) | 4·42 (1·68) | 4·12 (1·63) | 3·64 (1·52) | 3·43 (1·73) | 2·82 (1·05) |
| **IC**  H=1·30 (0·09) | 1·44 (0·18) | 1·45 (0·23) | 1·46 (0·17) | 1·56 (0·21) | 1·80 (0·35) | 1·66 (0·34) | 1·46 (0·10) | 1·58 (0·20) | 1·51 (0·14) |
| **Trunk pitch* (°)**  H=4·03 (1·85) | 16·1 (9·8) | 27·5 (13·1) | 13·5 (8·8) | 17·7 (7·6) | 34·0 (19·6) | 19·0 (10·8) | 18·6 (11·3) | 31·4 (10·9) | 13·8 (10·8) |
| **Trunk roll (°)*** H = 9·1 (4·3) | 28·2 (10.6) | 13·8 (9·2) | 30·7 (16·7) | 30·0 (12·6) | 18·5 (8·9) | 33·8 (18·6) | 27·0 (12·0) | 15·7 (9·9) | 27·5 (10·4) |
| **Trunk yaw (°)** H = 4·4 (1·3) | 17·2 (10·3) | 15·7 (12·7) | 15·6 (9·0) | 19·2 (8·6) | 20·2 (11·5) | 20·0 (11·0) | 17·1 (11·0) | 14.0 (6·0) | 14·1 (8·4) |
| **Trunk forward displacement (cm)**  H = 1·05 (0·49) | 6·6 (6·0) | 6·8 (6·5) | 3·7 (4·2) | 11·3 (7·0) | 11·3 (6·5) | 7·1 (6·4) | 8·5 (7·3) | 8·3 (8·2) | 5·2 (7·7) |
| **Shoulder plane angle (°)**  H = 9·74 (4·70) | 31·5 (7·8) | 31·4 (9·1) | 24·4 (8·1) | 39·3 (20·1) | 41·7 (19·4) | 37·9 (19·5) | 39·3 (20·1) | 41·7 (19·4) | 37·9 (19·5) |
| **Shoulder flexion (°)**  H = 14·1 (10·1) | 1·3 (21·8) | 6·1 (21·4) | 9·9 (22·5) | 15·5 (17·7) | 17·6 (18·9) | 17·5 (26·3) | 11·9 (26·2) | 18·4 (19·6) | 9·9 (26·0) |
| **Shoulder adduction* (°)**  H = 6·6 (13·5) | 30·4 (12·1) | 18·9 (14·3) | 21·1 (13·0) | 29·9 (13·0) | 26·4 (15·8) | 30·5 (15·9) | 28·6 (21·7) | 22·2 (16·0) | 24·7 (15·6) |
| **Elbow extension (°)**  H = 98·4 (12·5) | 97·6 (17·2) | 99·7 (15·0) | 94·6 (12·8) | 90·6 (13·9) | 91·9 (15·9) | 89·8 (11·6) | 97·7 (17·2) | 97·9 (20·9) | 92·6 (11·7) |
| **Wrist adduction* (°)** H = 25·1 (10·1) | 28·6 (14·0) | 23·6 (12·5) | 24·5 (12·8)** | 22·4 (10·8) | 24·1 (11·2) | 18·4 (6·0)** | 23·5 (11·0) | 30·3 (14·0) | 33·2 (13·2)** |

**Table S2. Range of elbow extension (mean (standard deviation) in degrees) for reaches to each target of the Test Task for both training groups at each time period (Pre, Post, Follow-up). Reaching was done without restricting the range of elbow extension in both groups.**

| **Training group** | **Restricted training group** | | | **Non-Restricted training group** | | |
| --- | --- | --- | --- | --- | --- | --- |
|  | PRE | POST | FOLLOW-UP | PRE | POST | FOLLOW-UP |
| **Target** |  |  |  |  |  |  |
| **Near Central** | 89·51 (12·84) | 88·22 (14·20) | 87·11 (12·20) | 87·55 (13·83) | 86·74 (15·58) | 85·50 (13·58) |
| **Far Central** | 99·35 (16·30) | 101·26 (16·73) | 99·84 (14·10) | 91·71 (11·85) | 95·04 (15·79) | 91·61 (11·81) |
| **Contralateral** | 98·72 (16·83) | 98·92 (18·14) | 96·65 (14·53) | 91·39 (13·28) | 93·57 (15·81) | 89·76 (11·64) |
| **Ipsilateral** | 101·48 (14·50) | 105·69 (17·59) | 104·74 (15·54) | 95·08 (13·68) | 98·80 (17·13) | 93·39 (10·45) |
